# What can the machine teach us?

**DOI:** 10.1186/s40635-025-00775-3

**Published:** 2025-07-07

**Authors:** Edward J. Schenck

**Affiliations:** 1 NewYork-Presbyterian Hospital, Weill Cornell Medicine, 1300 York Avenue, Box 96, New York, NY 10065 USA; 2 Division of Pulmonary & Critical Care Medicine, Joan and Sanford I. Weill Department of Medicine, Weill Cornell Medicine, New York, NY USA

**Keywords:** Machine learning, Unsupervised clustering, Sepsis, Epidemiology

Consider a 74-year-old woman presenting to the hospital with a productive cough, fevers, and tachycardia. She has comorbid hypertension, diabetes and slurred speech with some swallowing difficulty since a stroke several years ago. During her initial work up, her white blood cell count is elevated with increased immature granulocytes and her platelet count is 130. Her serum sodium is 133 mmol/L, her albumin is 25 g/L and her international normalized ratio is 1.5. The remainder of her routine chemistries are normal. Despite tachycardia and tachypnea, her mean arterial blood pressure is normal, and her peripheral oxygen saturation is 98% while breathing ambient air. Hazy bibasilar opacities are seen on a frontal chest radiograph, and after treatment with antibiotics, her tachycardia and tachypnea improve. She is admitted to the medical wards. Later that night she is found cool and pulseless.

What caused her death? She almost certainly had pneumonia, and this is communicated to her bereaved family. For epidemiologists, there is a fundamentally different questions to be answered. Was her death due to sepsis, “life-threatening organ dysfunction caused by a dysregulated host response”? [1]. She had an infection, her immune response was not optimal, and her life was not only threatened but ended by this same process. By many measures of account, however, the tally of her death would not be ascribed to sepsis [2]. Her experience and outcome would not be addressed by research and administrative efforts to improve the management of sepsis.

Sepsis is defined using arbitrary thresholds of organ function and lab abnormalities [1–3]. The true number of consequential infections is unknown. There is organ injury that is not captured by current approaches. Are patients with lab abnormalities just below a threshold fundamentally different than those just above? Novel methods are needed to address this concern. Li and colleagues used a combination of machine learning and classical epidemiology to add clarity to this phenomenon [4]. They first extracted a comprehensive electronic health record (EHR) data set representing a large multicenter cohort of patients admitted to an ICU in Canada. They then used this data set to evaluate whether there are groups of patients with potential sepsis that are not characterized by current frameworks, specifically the Center for Disease Control Adult Sepsis Events (ASE) [5]. They then developed a comprehensive pipeline of feature selection followed by dimension reduction and unsupervised clustering approaches. Concluding that the patient clustering derived from Robust and Sparse K-Means Clustering (RSKC) best represented the distribution of data, they then compared the distribution of patients meeting ASE criteria across clusters. Of 48 RSKC derived distinct clusters, 11 clusters contained greater than 50% of patients that met ASE criteria. Within these 11 clusters, those that were ASE—had an in-hospital mortality of 25.8%. Moreover 34.9% of the ASE—patients in the ASE majority clusters met a more liberalized sepsis criterion. Put another way, there are groups of ICU patients that are clinically similar to patients labeled as having sepsis but are currently missed. There are certain limitations to this work. They restricted their study to an ICU cohort, and it is not clear that ASE—patients meeting the more liberalized sepsis definition had an infection with consequence. More granular patient level epidemiology is needed. Lastly, the group relied on expert adjudication for feature selection that may have constrained the novelty of the derived clusters.

The implications of this work are self-evident. Sepsis definitions are arbitrary, and it follows that the resultant epidemiology has significant gaps. We cannot improve what we do not measure. This work clearly shows that data can guide discovery. Unsupervised learning has the potential to teach us something new. Hopefully this innovative work will lead us closer to a more holistic understanding of infection and the human condition.

## Data Availability

This work involves no primary data.
